# What Is Congelation? Etc.

**Published:** 1859-07

**Authors:** 


*What is Congelation?* etc. By R. E. HARRISON. 16mo. pp. 90, 1858: London.

*On the Loss of Teeth*, etc. By THOS. HOWARD. 16mo., pp. 61. 1858: London.

The above titles refer to two little books which very creditably represent a large class of dental publications. They are simply expensive but possibly profitable advertisements in the guise of popular treatises. The profession will find them of little or no value. Books of this kind, as far as we have been able to examine, are made up chiefly of extracts from the more systematic and scientific treatises upon which the student should rely in pursuing his studies.

